# Detection of *Trypanosoma cruzi* in white-eared opossums (*Didelphis albiventris*) from Canoinhas, Santa Catarina State, Brazil^1^

**DOI:** 10.1590/S1984-29612025003

**Published:** 2025-02-03

**Authors:** Giane Helenita Pontarolo, Daniela Pedrassani, Luís Felipe Kühl, Monique Paiva Campos, Thais Cristina Tirado, Fabiano Borges Figueiredo, Thállitha Samih Wischral Jayme Vieira, Ana Cláudia Calchi, Marcos Rogério André, Rafael Felipe da Costa Vieira, Ivan Roque de Barros

**Affiliations:** 1 Departamento de Medicina Veterinária, Universidade Federal do Paraná – UFPR, Curitiba, PR, Brasil; 2 Departamento de Medicina Veterinária, Universidade do Contestado – UnC, Canoinhas, SC, Brasil; 3 Laboratório de Referência em Leishmanioses, Instituto Carlos Chagas – ICC, Fundação Oswaldo Cruz – Fiocruz, Curitiba, PR, Brasil; 4 Department of Chemistry, The University of North Carolina at Charlotte, Charlotte, USA; 5 Vector-Borne Bioagents Laboratory – VBBL, Departamento de Patologia, Reprodução e Saúde Única, Faculdade de Ciências Agrárias e Veterinárias – FCAV, Universidade Estadual Paulista – UNESP, Jaboticabal, SP, Brasil; 6 Department of Epidemiology and Community Health, The University of North Carolina at Charlotte, Charlotte, USA; 7 Center for Computational Intelligence to Predict Health and Environmental Risks – CIPHER, The University of North Carolina at Charlotte, Charlotte, USA

**Keywords:** Trypanosoma cruzi, Chagas disease, Didelphis, Trypanosoma cruzi, doença de Chagas, Didelphis

## Abstract

Opossums are synanthropic animals that participate in the zoonotic transmission cycles. Chagas disease, a neglected tropical disease caused by *Trypanosoma cruzi*, affects many domestic and wild animals and humans worldwide. This study aimed to determine the occurrence of *T. cruzi* in free-ranging opossums in Canoinhas, Santa Catarina, Brazil. Fifty opossums (*Didelphis albiventris*) (33 captured and 17 road-killed) were evaluated using Nested-PCR assay. All tissue samples were negative (0/17). Eight of the 33 (24.24%; 95% CI:11.94–40,89%) blood samples were positive for *T. cruzi*. No significant associations were found between the sex (male/ female, p = 0.423), the trap area (rural/urban, p = 0.163), and positivity for *T. cruzi* in opossum blood samples. All samples showed 100% identity with *T. cruzi* (KF788250) isolated from *Panstrongylus megistus* in São Paulo, Brazil. The phylogenetic analysis model allocated all sequences obtained from *D. albiventris* to the large TcI clade of *T. cruzi.* This study provides the first record of *T. cruzi* in white-eared opossums in Canoinhas, Santa Catarina, southern Brazil.

## Resumo

Gambás são animais sinantrópicos que participam do ciclo de transmissão dessa zoonose. Doença de Chagas é uma doença tropical negligenciada, causada por *Trypanosoma cruzi*, ocorre mundialmente em animais domésticos, silvestres e seres humanos. O objetivo deste estudo foi determinar a ocorrência de *Trypanosoma cruzi* em gambás de vida livre no município de Canoinhas, estado Santa Catarina, Brasil. Para isso, 50 gambás (*Didelphis albiventris*) (33 capturas e 17 necropsias) foram avaliados por ensaio de Nested-PCR. Todas as amostras de tecido foram negativas (0/17). Oito de 33 (24.24%; 95% CI:11.94-40,89%) amostras de sangue foram positivas para *T. cruzi*. Não foi encontrada associação significativa entre o sexo (macho/fêmea, p = 0.423), área de captura (rural/urbana, p = 0.163), e positividade para *T. cruzi* em amostras de sangue dos gambás. Todas as amostras positivas demonstraram 100% identificação com *Trypanosoma cruzi* (KF788250), isolado de *Panstrongylus megistus* em São Paulo, Brasil. O modelo de análise filogenética alocou todas as sequências obtidas de *D. albiventris* no clado TcI de *T. cruzi*. Este estudo fornece o primeiro relato de *T. cruzi* em gambás de orelha branca em Canoinhas, Santa Catarina, sul do Brasil.

## Introduction

*Trypanosoma cruzi* is the etiological agent of Chagas disease (CD) in humans, and affects humans as well as wild and domestic mammals in endemic areas of Latin American countries. *Trypanosoma cruzi* subpopulations are further subdivided into seven distinct taxonomic units (DTUs): TcI–TcVI and Tcbat (Marcili et al., 2009; Zingales et al., 2012). TcI is the most abundant and widely dispersed *T. cruzi* DTUs in South, Central, and North America and is associated with chagasic cardiomyopathy. Moreover, it is observed in triatomine vectors and sylvatic hosts, such as the genus *Didelphis,* and is associated with the sylvatic and domestic cycles of CD (Zingales et al., 2012).

The white-eared opossum is a marsupial, opportunistic, and omnivorous animal characterized as a highly synanthropic species because of its ability to adapt to urban and devastated areas and its agile nocturnal and nomadic climbing. Demonstrated to be a competent bioaccumulator of TcI diversity in Brazil, with high infectivity potential and high levels of parasitemia (Jansen et al., 2018).

CD affects poor rural populations and causes subclinical infections, cardiac and digestive syndromes, and even death. Transmission of *T. cruzi* can occur via transfusion of infected blood, organ transplantation, ingestion of contaminated food, congenital transmission, or contact with the feces of the triatomine bugs (WHO, 2013).

The Canoinhas municipality comprises a large forest area and abundant wildlife and is thus a favorable environment for the maintenance of zoonotic agents and possible spillover. The detection of *T. cruzi*, the etiological agent of CD in humans, constitutes important epidemiological data because opossums are considered wild reservoirs of this pathogen. Accordingly, this study aimed to determine the occurrence and molecular characteristics of *T. cruzi* in white-eared opossums from Canoinhas, Santa Catarina State, southern Brazil.

## Material and Methods

### Study area

The study was conducted in the Canoinhas municipality (50° 23' 25” W, 26° 10' 38” S). Canoinhas is located in the northern region of Santa Catarina State, southern Brazil, characterized by semideciduous Atlantic Forest fragments, and has a temperate climate with an annual average temperature of 17 °C.

### Sampling and DNA extraction

The samples were retrieved according to the method described in a previous study by Pontarolo et al. (2021). Fifty white-eared opossums (29 were females and 21 males) were captured in rural (20) and urban (30) areas of Canoinhas municipality, using Tomahawk traps baited with fruit. Sampling was performed by spontaneous demand of the Environmental Military Police of Canoinhas municipality and based on the report of the occurrence of opossums in human dwellings. A total of 589 trap-night (number of trap * number of days) yielded 33 captures, with a success rate of 6.92% in the rural area (20/289) and 4.33% in the urban area (13/300). Additionally, 17 road-killed opossums were evaluated. Geographical coordinates from the location of the sampled opossums were recorded (GPSMAP^®^ 64 series, Garmin^®^ International Inc., KS, USA).

After chemical restraint, opossums were identified with ear tagging. Subsequently, EDTA blood samples were collected and stored at -20 °C until molecular analysis. After the procedures, opossums were monitored and later released at the place of capture. Fragments of spleen (n = 15) and liver (n = 2) tissues were collected from road-killed opossums and stored at –20 °C until molecular analysis (Pontarolo et al., 2021).

DNA was extracted from whole blood or tissue samples of fifty white-eared opossums using a commercial kit (DNeasy^®^ Blood & Tissue, Qiagen, Hilden, Germany), according to the manufacturer’s instructions for the semi-automated DNA extraction platform (Qiacube, Qiagen^®^, Germany). Ultrapure water was used in parallel as a negative control to monitor cross-contamination. The concentration of the extracted DNA was evaluated by fluorimetry using a Qubit^®^ dsDNA HS Assay (Qubit^®^ 2.0 Fluorometer, Invitrogen, CA, USA).

### Nested Polymerase Chain Reaction (nested PCR) assays

To ensure successful DNA extraction, PCR for the endogenous mammalian gene glyceraldehyde-3-phosphate dehydrogenase (*gapdh*) (Birkenheuer et al., 2003), was performed using all samples. DNA samples were further screened using a previously described nested PCR assay targeting the ssrRNA gene nucleus of trypanosomatids (Noyes et al., 2000) with some modifications. Briefly, external primers TRY927F (5’-GAAACAAGAAACACGGGAG-3’) and TRY927R (5’-CTACTGGGCAGCTTGGA-3’) were employed for the first round, and internal primers SSU561FT (5’-GGGATAACAAAGGAGCA-3’) SSU561R (5’-CTGAGACTGTAACCTCAAAGC-3’) were used for the second round.

DNA amplification using PCR was performed in a final volume of 25 μL, containing 12.5 μL of master mix (Amplitaq gold 360, ThermoFisher^®^, Massachusetts, USA), 2.5 μL of enhancer, 1 μL of each primer (20 pmol), 3 μL of water, and 5 μL of DNA. The products from the first round using primers TRY816F:R were diluted at a ratio of 10:1 in water, and 1 μL was used as a template in the second round under the same conditions with primers SSU561F:R. The cycling conditions were as follows:95 °C for 10 min, followed by 30 cycles of 94 °C for 30 s, 55 °C for 60 s, 72 °C for 90 s, and a final extension at 72 °C for 10 min. As positive controls for the reaction, 10 ng/μL of the *Leishmania* sp. (MHOM/BR/1975/M2903) and the *T. cruzi* strain 42CT were used.

### Sequencing and phylogenetic analysis

The amplicons obtained from eight *Trypanosoma*-positive samples were sequenced in both directions using the Sanger method (Sanger et al., 1977). Partial nucleotide sequences of the ssrRNA gene of *Trypanosoma* have been deposited in the GenBank^®^ database (accession numbers: OQ726372, OQ726373, OQ726374, OQ726375, OQ726376, OQ726377, OQ726378, and OQ726379).

The obtained sequences were subjected to a quality screening test using Phred-Phrap software (version 23) (Ewing & Green, 1998) to evaluate the quality of the electropherograms and obtain consensus sequences by aligning the sense and antisense sequences. The BLASTn program (Altschul et al., 1990) was used to compare the nucleotide sequences obtained with those previously deposited in the GenBank database (Benson et al., 2002). The sequences saved in “FASTA” format were aligned with other homologous sequences of each agent retrieved from the database (Genbank) using the Mafft software (Katoh & Standley, 2013) and edited via Bioedit v. 7.0.5.3 (Hall, 1999). W-IQ-Tree software was used to choose the evolutionary model based on the Bayesian Information Criterion (BIC), and phylogenetic analysis was carried out using the maximum likelihood method (Trifinopoulos et al., 2016). Clade support indices were evaluated using bootstrap analyses (Felsenstein, 1985) with 1000 repetitions. The editing of phylogenetic trees and rooting (via the outer group) were performed using TreeGraph 2.0.56-381 beta software (Stöver & Müller, 2010).

### Statistical analyses

Fisher’s exact test was used to determine the differences between individual factors (sex and trap area) and those associated with positivity for *T. cruzi*. Odds ratios (OR), 95% confidence intervals (CIs), and p-values were calculated for each variable, with results considered significant at p < 0.05. Data were analyzed using GraphPad Prism (version 6).

## Results

Eight of the 50 (16%; 95% CI:7.17-29.11%) white-eared opossums tested positive for *T. cruzi* using nested PCR. All tissue samples were negative (0/17). The endogenous mammalian *gapdh* gene was consistently amplified in all samples.

The *T. cruzi* in white-eared opossums was observed only in blood samples. Eight of the 33 (24.24%; 95% CI:11.94–40,89%) blood samples tested positive for *T. cruzi* using nested PCR. Seven of the eight (87.5%) positive animals were female and were found in the rural area. Though no significant associations were found, opossums in rural areas are approximately 6.43 times more likely to be infected by *T. cruzi* compared to those in urban areas (p = 0.163), and females are approximately 3.93 times more likely to be infected by *T. cruzi* compared to males (p = 0.423, Table 1).

**Table 1:** Occurrence of the *Trypanosoma cruzi* in blood samples of opossums in Canoinhas, Santa Catarina State, southern Brazil, 2023.

**Variable**		**+/n**	**OR (95% CI)**	**p-value**
Sex	Female	7/23 (30.43)	3.93 (0.41 – 37.31)	0.423
	Male	1/10 (10.00)		
Trap Area	Rural	7/20 (35.00)	6.43 (0.68 – 60.53)	0.163
	Urban	1/13 (7.69)		

+ number of positive animals; n: number of analyzed samples; OR: ODDS ratio; 95% CI: 95% confidence interval.

**Figure 1:**
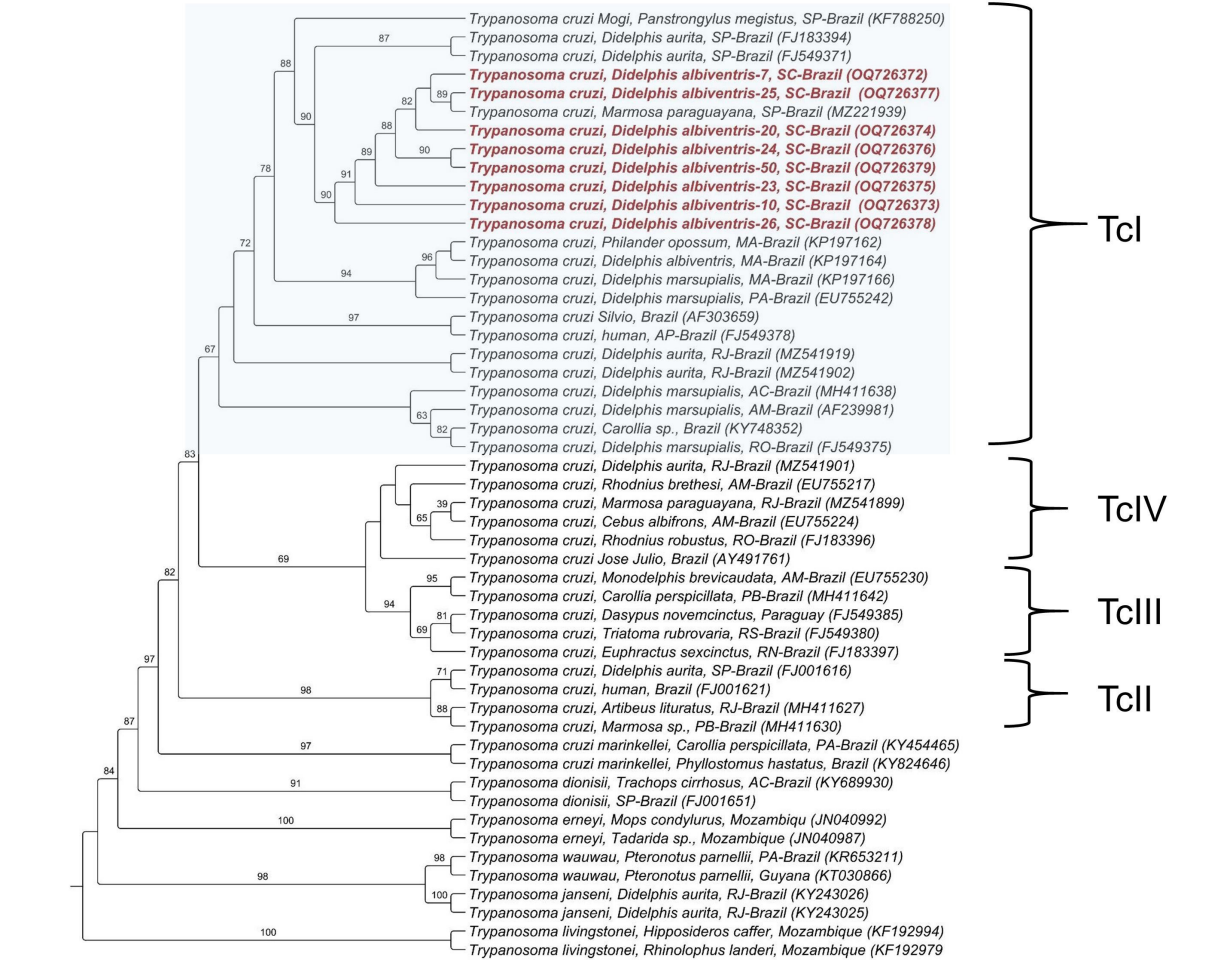
Evolutionary analysis using the maximum likelihood method. Maximum likelihood phylogenetic analysis was performed with an 870 bp alignment, and the TIM3+G evolutionary model allocated all sequences obtained from *D. albiventris* to the large TcI clade of *T. cruzi*. The sequences formed a subclade together with a sequence detected in *Marmosa paraguayana* from São Paulo, Brazil, with a bootstrap of 90%.

DNA sequencing of all samples showed 100% identity with *T. cruzi* (KF788250) isolate from *Panstrongylus megistus* in São Paulo State, southeastern Brazil. An evolutionary analysis using the maximum likelihood method is shown in Figure 1. The phylogenetic analysis model allocated all sequences obtained from *D. albiventris* to the large TcI clade of *T. cruzi*.

## Discussion

Our results show that the occurrence of *T cruzi* in white-eared opossums was observed in 24.24% (8/33) of blood samples in this study, reiterating that blood collection is an efficient methodology for monitoring the *T. cruzi* presence in wild reservoirs. All tissue samples were negative (0/17).

A previous study using nested PCR on DNA extracted from blood clots demonstrated that this method is sensitive and suitable for evaluating the diversity of trypanosomes infecting sylvatic mammals, including marsupials (Rodrigues et al., 2019). A molecular diagnostic study using nested PCR in blood samples of opossums (*Didelphis albiventris)* from Campo Grande, Mato Grosso do Sul State, Brazil, observed that 32.5% (13/40) of the opossums were infected with *T. cruzi* (Nantes et al., 2019). The combined prevalence of *T. cruzi* infection using PCR was 36.4% (95% CI, 7.9 - 64.8%) in *D. albiventris* in a rural area in humid Chaco, Argentina (Alvarado-Otegui et al., 2012).

We statistically evaluated the blood sample results obtained in this preliminary study. No significant association was found between the sex (male/ female, p = 0.423), the trap area (rural/urban, p = 0.163), and positivity for *T. cruzi* in opossum blood samples, indicating that infection is widespread in this opossum population, regardless of these variables.

Our study showed that the DNA sequences from all *Trypanosoma*-positive samples shared 100% identity with *T. cruzi* (KF788250) isolated from *P. megistus* in São Paulo, Brazil. The phylogenetic analysis model allocated all sequences obtained from *D. albiventris* to the large TcI clade of *T. cruzi,* consistent with the known distribution of this clade in Brazil. *Panstrongylus megistus*, a sylvatic triatomine, harbors the TcI sylvatic strain, which is the most abundant and widely dispersed DTU in America (Zingales et al., 2012; Martins et al., 2014).

CD is a neglected disease typically observed in rural regions, where the cycle is complete, with the presence of the vector, species of triatomines, reservoirs, and wild animals, including opossums, and humans. However, urban transmission patterns involve oral transmission through contaminated foods (Brasil, 2019).

In Santa Catarina there are no reports of confirmed cases of domiciled vector transmission; triatomine bugs are observed only in their wild form but are not implicated in the transmission chain to humans. The wild vectors *P. megistus, Rhodnius domesticus*, and *T. tibiamaculata* are involved in maintaining the wild cycle of *T. cruzi* and are observed in Santa Catarina (DIVE, 2021).

The white-eared opossum uses tree hollows, wood piles, palm crowns, and other locations for shelter, thus being exposed to *T. cruzi* transmission cycles occurring in the wild. (Jansen et al., 2018). This behavior increases the probability of encounters between opossums and triatomines, consequently enhancing *T. cruzi* transmission chances.

The opossums were able to establish chronic infections, and in experimental infections, it was possible to observe that they were positive for a long period in blood cultures (Jansen et al., 1997). They displayed a high infectivity potential, mainly for TcI, with high levels of parasitemia (Jansen et al., 2018). This could explain the high positivity rate in the blood in this study.

## Conclusions

In conclusion, this study presents the first record of *T. cruzi* in white-eared opossums in Canoinhas, Santa Catarina, southern Brazil, revealing a 24.24% prevalence with no significant associations between sex or capture area, indicating that *T. cruzi* is widespread in this opossum population and highlighting the need for ongoing monitoring in wild reservoirs, particularly as the identified strains belong to the TcI clade and exhibit genetic homogeneity with isolates from other regions of Brazil.
